# Renal function and blood pressure in 11 year old children born extremely preterm or small for gestational age

**DOI:** 10.1371/journal.pone.0205558

**Published:** 2018-10-12

**Authors:** Maria Vollsæter, Thomas Halvorsen, Trond Markestad, Knut Øymar, Per Magne Ueland, Klaus Meyer, Øivind Midttun, Anne-Lise Bjørke-Monsen

**Affiliations:** 1 Department of Pediatrics, Haukeland University Hospital, Bergen, Norway; 2 Department of Clinical Science, University of Bergen, Bergen, Norway; 3 Department of Pediatrics, Stavanger University Hospital, Stavanger, Norway; 4 Laboratory of Clinical Biochemistry, Haukeland University Hospital, Bergen, Norway; 5 Bevital A/S, Armauer Hansens Hus, Bergen, Norway; Centre Hospitalier Universitaire Vaudois, FRANCE

## Abstract

**Background:**

Preterm birth and low birth weight are associated with reduced nephron numbers and increased risk of hypertension and kidney disease in later life.

**Aims:**

We tested the hypothesis that extremely preterm birth and intrauterine growth restriction is associated with decreased renal function in mid childhood.

**Methods:**

At 11 years of age the following measures were obtained in a regional cohort of children born extremely premature (EP, i.e. < 28 weeks gestational age—GA) or with extremely low birth weight (ELBW, i.e. BW < 1000 grams) and in matched controls born at term with appropriate BW (AGA): Height, weight, abdominal circumference, triceps and subscapular skin fold thicknesses, blood pressure, plasma levels of creatinine, cystatin C and symmetric dimethyl arginine (SDMA). Small for gestational age (SGA) was defined as a BW < 10^th^ percentile for GA. Glomerular filtration rate (GFR) was estimated according to the equations by Schwartz, Zappitelli and Gao.

**Results:**

Fifty-seven of 61 eligible EP/ELBW children, 20 (35%) born SGA, and 54 controls, were assessed. Estimated GFR decreased while plasma SDMA increased from the children born AGA at term through those born preterm AGA to preterm SGA. Systolic BP was correlated to fat mass indices (p<0.03), but not to renal function (p>0.2) and did not differ between the groups.

**Conclusions:**

Children born EP/ELBW, particularly those born SGA, had impaired renal function at age 11 years as judged from estimated GFRs and plasma levels of SDMA. Since reduced renal function is associated with an increased risk of later disease, these children should be followed in order to minimize additional risk factors.

## Introduction

Preterm birth implies short- and long-term consequences for most organ systems. The fetus might have been subject to adverse intrauterine development leading to preterm birth [1], and thereafter has to finalize growth and development outside the uterus, often in a neonatal intensive care unit, exposed to a range of internal and external stressors with the potential to induce harm [2]. Remarkable improvements in survival for preterm born children the last few decades have resulted in large cohorts of long-term survivors. Their health is of increasing public relevance [3, 4], as late morbidity might be increasing in these populations as they grow older. This calls for research into long-term risks in preterm populations.

For the renal system, being born preterm, with low birth weight (LBW, i.e. < 2500 gram) or small for gestational age (SGA, i.e. BW < 10^Th^ percentile for GA), is associated with smaller kidneys, reduced nephron numbers, possibly morphologically abnormal nephrons and reduced glomerular filtration rate [5]. This implies an increased risk of developing hypertension (HTN) and chronic kidney disease (CKD) in later life [5–9]. Thus, early diagnosis of renal dysfunction is warranted in order to minimise additional risk factors for CKD [10].

The gold standard for assessment of renal function is *glomerular filtration rate (GFR)* based on the clearance of the exogenous marker inulin, but also clearance of iothalamate and iohexol are used [11]. While these methods are expensive, time consuming and associated with a risk of ionizing radiation, estimated GFR based on the endogenous markers creatinine or cystatin C, either alone or in combination, is easier and more commonly used [12]. In children, there are several GFR equations that include height, weight, age and gender [13], e.g. the bedside Schwartz equation and the quadratic formula by Gao, both based on serum creatinine, and the Zappitelli equation, based on both serum creatinine and cystatin C [14–16].

C*reatinine*, the waste product from creatine and phosphocreatine, found mainly in skeletal muscle, is freely filtered through the glomerulus and secreted through the proximal tubules. Its value as a renal function marker is hampered by biological and analytical variations. There is considerable variation in serum levels due to differences in height, muscle mass, gender and diet [17], the analytical methods show substantial variability, and there is no standardized test method [18]. *Cystatin C* is produced at a constant rate in all cells, and excreted by glomerular filtration followed by catabolism in the tubular cells. As it is less affected by height, weight, muscle mass and diet than creatinin, its plasma concentration has been proposed as a better renal function marker for pediatric patients [19]. *Symmetric dimethylarginine (SDMA)* is a methylated arginine metabolite produced in all nucleated cells and excreted in the kidneys. The plasma level is correlated to inulin clearance in children, and thus, SDMA has been put forward to serve as an endogenous marker of renal function [20].

We investigated renal function by creatinine, cystatin C, SDMA and estimated GFR in two groups of 11 year old children; term-born children with appropriate birth weight (BW) for gestational age (AGA) and children born extremely preterm (EP) or with an extremely low birth weight (ELBW). Our hypothesis was that being born EP/ELBW and especially SGA within the EP/ELBW group, was associated with decreased renal function.

## Methods

### Study population

As part of a prospective national study [21], a cohort of infants born EP, i.e. gestational age (GA) <28 weeks, or with ELBW, i.e. BW <1000 grams, within Western Norway Health Authority in 1999 and 2000, were prospectively recruited for follow-up at birth. The health region serves a population of approximately 1.1 million, and all the infants were initially treated at the university hospitals in Bergen or Stavanger.

The children were invited for follow-up at 11 years of age. For each EP/ELBW participant, the next-born child of the same gender with GA >37 weeks and BW >3000 grams was identified from birth protocols in the maternity ward and invited as a control. If the parents declined, the next-born child was approached until a match was obtained.

Neonatal data were obtained from compulsory notifications to the Medical Birth Registry of Norway and from registration forms developed for the study and completed by neonatologists during the stay in the neonatal intensive care nursery, and by pediatricians and parents during follow-up [22]. Small for gestational age (SGA) was defined as BW <10^th^ percentile for GA according to Norwegian growth curves [23].

The Regional Committee on Medical Research Ethics granted ethical approval of the protocol, and the mothers gave written, informed consent.

### Data collection and biochemical analysis

At 11 years of age (in 2010–2011), the children were assessed at the University Hospitals in Bergen or Stavanger, according to place of birth. Height, weight, waist circumference, triceps and scapular skinfolds were measured. Body mass index (BMI: weight/height^2^) and subscapular to triceps skinfold ratio (STR) were calculated as estimates of body fat deposition and truncal fat mass [24]. Systolic and diastolic blood pressures (BP) were measured three times by an electronic device (GE Critikon Dinamap XL vital signs) and a mean value was calculated.

Blood samples were obtained by antecubital venipuncture and collected into EDTA Vacutainer Tubes (Becton Dickinson) for separation of plasma and in Vacutainer Tubes without additives (Becton Dickinson) for separation of serum. Blood samples for preparation of EDTA-plasma were placed in ice water, and plasma was separated within 4 hours. The samples were stored at –80°C until analysis.

Renal function markers included plasma creatinine, cystatin C, symmetric dimethylarginine (SDMA) and estimated GFR. Plasma levels of creatinine and SDMA were measured using liquid chromatography-tandem mass spectrometry (LC-MS/MS) [25]. Creatinine measured by LC-MS/MS is about 10% lower than values obtained with a creatinine iminohydrolase procedure. The difference may be related to the higher specificity of and less interference for the LC-MS/MS method. The lower limit of detection (LOD) of creatinine in this assay is 0.25 μmol/L, within-day CV is 3–5%, and between-day CV is 2–6% (www.bevital.no). Plasma levels of cystatin C were measured using immuno-MALDI-MS, with a LOD of 0.003 μg/mL and a within-day CV of 4–6% (www.bevital.no) [26].

Estimated GFR was based on the bedside Schwartz equation, i.e. 0.413*height/Creatinine, the Zappitelli equation, i.e. (507.76 × e^0.003×height^)/(Cystatin C^0.635^ × Creatinine^0.547^) [15, 16] and the new quadratic formula by Gao et al, i.e. 0.68 x (height (cm)/serum Creatinine (mg/dl))-0.0008 x (height(cm)/serum Creatinine (mg/dl))^2^ + 0.48 x age (years)—(21.53 in males or 25.68 in females) [14].

### Statistical analysis

Results are presented as means, standard deviations and 95% confidence intervals (CIs) or as medians and interquartile range (IQR) and compared by ANOVA/Student’s t-test or Kruskal Wallis test. Differences in categorical variables were tested by Chi-Square tests. Trends for differences according to birth status (from term-born via preterm AGA to preterm SGA) were assessed by linear regression. The Spearman’s test was used to calculate correlations, and multiple linear regressions to assess renal function after adjusting for birth status and growth parameters (AGA vs. SGA).

The SPSS statistical package (version 23) was used for the statistical analyses and two-sided p-values < 0.05 were considered statistically significant.

## Results

### Demographics (Table 1)

Fifty-seven eligible EP/ELBW children were examined at a mean age of 11.4 (SD 0.4) years (Table 1). The GA ranged from 24 to 31 weeks and BW from 450 to 1250 grams; 46 (81%) were EP, 49 (86%) were ELBW. Twenty (35%) were born SGA; their mean BW was 200 grams lower than for those born AGA (p<0.001) despite a 2 weeks higher mean GA (p<0.001). Blood for assessment of renal function was successfully obtained from 50/57 EP/ELBW and from 45/54 control children, with no differences as regards other measurements between those with or without blood samples.

**Table 1:** Demographic characteristics in the term-born and preterm-born children (n = 111).

Variables	Term-born AGA children (n = 54)	Preterm-born children		P value	P for trend††
		AGA (n = 37)	SGA (n = 20)		
**At birth**					
Weight, g	3701 (3582, 3819)	918 (867, 968)	724 (657, 791)	<0.001*	
Gestational age, wks		26.1 (25.7, 26.5)	28.0 (27.2, 28.7)	<0.001*	
Male sex, n (%)	29 (54%)	16 (43%)	13 (65%)	0.27**	
**At study inclusion**					
Age, y	11.7 (11.2–12.0)	11.4 (11.1–11.8)	11.3 (11.0–11.8)	0.08†	
Weight, kg	41.3 (38.9, 43.6)	40.1 (37.5, 42.6)	36.2 (32.0, 40.3)	0.07*	0.03
Height, cm	151.5 (149.2, 153.8)	149.2 (146.7, 151.8)	142.6 (139.7, 145.4)	<0.001*	<0.001
BMI (kg/m^2^)	17.8 (17.1, 18.6)	17.8 (17.1, 18.6)	17.6 (16.2, 19.1)	0.95*	0.81
Waist circumference, cm	66.3 (64.1, 68.5)	65.9 (63.1, 68.7)	65.1 (61.0, 69.3)	0.86*	0.59
Triceps skinfold, mm	12.3 (11.2, 13.5)	11.7 (10.2, 13.2)	11.1 (9.1, 13.1)	0.53*	0.26
Subscapular skinfold, mm	8.6 (7.3, 9.8)	8.7 (7.3, 10.1)	9.1 (7.0, 11.2)	0.11*	0.66
Subscapular to triceps ratio	0.70 (0.64, 0.76)	0.76 (0.69, 0.82)	0.82 (0.68, 0.97)	0.10*	0.03
Systolic BP, mm HG	109.1 (106.7, 111.4)	113.0 (109.9, 116.2)	110.4 (106.2, 114.6)	0.12*	0.28
Diastolic BP, mm HG	64.1 (62.1, 66.1)	64.8 (62.1, 67.4)	63.8 (59.9, 67.6)	0.97*	0.98

The results are presented as mean (95% CI), comparison by Anova test*,

as number (%), comparison by Pearson Chi-Square test**

and median and IQR, comparison by Kruskal Wallis test†.

P for trend by linear regression††.

AGA = appropriate birthweight for gestational age, SGA = small birthweight for gestational age.

At 11 years mean height was lower in the EP/ELBW children, whereas mean weight and BMI were similar to that of the term controls. Within the EP/ELBW children, those born SGA were shorter, but their mean weight and BMI were similar to that of those born AGA. STR skinfold thickness increased from the term-born group through the preterm AGA and preterm SGA children (Table 1).

There were no differences in mean systolic or diastolic BP between term and preterm AGA or SGA children (Table 1). Systolic BP correlated positively with BMI, weight, waist circumference, subscapular skinfold and STR (r>0.22, p<0.03), but not with height, triceps skinfold or renal function parameters.

### Renal function

All renal function parameters were inter-correlated (Table 2). The SDMA level increased and the estimated GFR decreased from the term-born group through the preterm AGA and preterm SGA groups, whereas there were no significant group differences for creatinine or cystatin C levels (Table 3).

**Table 2:** Spearman correlation between renal function parameters in term-born and preterm-born children at 11 years (n = 95).

	Creatinine	Cystatin C	SDMA	GFR Schwartz	GFR Zappitelli
Cystatin C, μmol/L	0.33**				
SDMA, μmol/L	0.40*	0.39*			
GFR Schwartz	-0.88*	-0.28**	-0.41*		
GFR Gao	-0.85*	-0.28*	-0.41*	0.96*	0.65*
GFR Zappitelli	-0.68*	-0.89*	-0.50*	0.66*	

P<0.001*,

P<0.01**

**Table 3:** Renal function parameters in the term-born and preterm-born children at 11 years (n = 95).

Renal function parameters*	Term-born AGA children (n = 45)	Preterm-born children	P*	P trend**	P†	P††
		AGA (n = 33)	SGA (n = 17)				
Creatinine, μmol/L	51.0 (49.2, 52.8)	51.6 (49.8, 53.5)	53.6 (49.5, 57.6)	0.34	0.17	0.31	0.37
Cystatin C, mg/L	0.86 (0.82, 0.90)	0.89 (0.84, 0.94)	0.91 (0.85, 0.98)	0.39	0.17	0.21	0.58
SDMA, μmol/L	0.55 (0.52, 0.57)	0.56 (0.53, 0.58)	0.61 (0.57, 0.66)	0.01	0.009	0.09	0.02
GFR Schwartz	110.0 (106.2, 113.7)	106.8 (103.3, 110.4)	99.0 (91.0, 106.9)	0.009	0.003	0.03	0.07
GFR Gao	105.6 (103.4, 107.8)	103.5 (101.4, 105.6)	98.4 (92.4, 104.4)	0.007	0.002	0.02	0.05
GFR Zappitelli	104.8 (100.3, 109.3)	101.2 (96.2, 106.3)	95.1 (89.7, 100.5)	0.055	0.017	0.05	0.13

The results are presented as mean (95% CI), comparison by Anova test*

and P for trend by linear regression**

and by Students’s t-test between † term and preterm

and between †† AGA and SGA preterms.

AGA = appropriate birthweight for gestational age, SGA = small birthweight for gestational age

After adjusting for age, gender, weight and height in a multiple linear regression model, there was an increase in plasma creatinine (B = 2.1, p = 0.02), cystatin C (B = 0.04, p = 0.05) and SDMA (B = 0.03, p = 0.009) and a reduction in GFR Schwartz (B = -4.4, p = 0.02), GFR Gao (B = -2.8, p = 0.02) and GFR Zappitelli (B = -5.8, p = 0.007) from the term-born through the preterm AGA and preterm SGA groups. The endogenous renal markers (including estimated GFR) did not correlate with systolic or diastolic BP (P>0.13).

## Discussion

At 11 years of age, children born EP/ELBW had impaired renal function compared to term born peers, as reflected in lower GFR and higher SDMA. Renal function was more impaired in children born preterm and SGA compared to those born preterm and AGA. Blood pressure was unrelated to GA, BW or SGA vs. AGA status, but systolic BP was positively associated with indices of increased fat mass.

### Renal function in relation to prematurity, low birth weight and SGA

There is increasing awareness that adverse intrauterine conditions may lead to developmental programming causing non-communicable diseases later in life, a concept labeled ‘developmental origins of health and disease’ (DOHaD). For hypertension and renal disease, these issues may be related to adverse fetal events that modify the nephron number and/or function [27].

Different research groups have reported higher blood pressure or reduced renal function in groups of individuals born preterm and/or at a low birth weight [7–9], corresponding to our findings. However, these previous studies have examined adult populations in a retrospective manner, and based their sample selection on reported birth weight less than 2500 gram [7–9], and thus may not be comparable. In an unselected population, BW less than 2500 gram principally corresponds to being born moderately preterm and AGA, or being born SGA at term. Applying this inclusion criterion, it may thus be challenging to disentangle if outcomes relate to preterm birth *per se* or to an adverse intrauterine environment. Moreover, studies investigating cohorts born at different gestational ages may not be directly comparable. Fetal kidney development will have reached different developmental stages at birth and the mechanisms for reduced kidney function are likely to differ. Finally, some studies have investigated only males [7], or found the association only in males (Li), whereas the distribution of gender in our cohort was approximately as would be expected in a preterm born group of individuals.

The mechanisms linking preterm birth, fetal growth restriction and impaired renal function are poorly understood. Nephrogenesis begins around the ninth week of pregnancy and terminates around 36 weeks’ gestation in term-born infants. More than 60% of adult nephrons are formed during the last two trimesters of pregnancy; thus, the number of nephrons correlates positively with GA at birth [28]. Nephrons have no re-generational ability if damaged, and must last a life-time. Preterm birth implies premature exposure to the haemodynamic transitions of birth, leading to the postnatal increase in systemic blood pressure and in renal blood flow, potentially impacting nephron development [29–33]. Preterm birth also implies exposure to a range of intensive care interventions that may have additional negative impact on nephron development and later renal function, such as prenatal steroids [34], the use of nephrotoxic drugs [35], exposure to infections and suboptimal nutrition. The association between prematurity and altered renal function may therefore be explained by both development of fewer nephrons after premature birth *per se*, as well as by abnormal nephrons that are formed postnatally, while the preterm infant is exposed to neonatal intensive care [28].

Fetal growth restriction *as such* may also result in lower numbers of nephrons in premature SGA infants, with the number of nephrons increasing by approximately 250.000 per kg birthweight [33, 36]. A reduced number of nephrons is an important factor in the development of later kidney disease and hypertension [30], a risk that seems to be amplified in those born SGA [29, 31, 32].

Thus, the present study reinforces concerns expressed previously for those born *both* prematurely *and* SGA [37, 38]. The finding that children born preterm and SGA have poorer renal function than children born preterm and AGA, suggests that disadvantageous antenatal factors are likely to be involved in the causal chain, in addition to the preterm birth *as such*. Nevertheless, it is important to bear in mind that all markers of renal function were within published normal reference ranges [15, 16, 19, 39, 40] in all groups at 11 years of age, and therefore the long-term significance of the findings are unknown.

### Hypertension in relation to prematurity, low birth weight and SGA

Hypertension is both a major cause and a result of chronic kidney disease. Both prematurity and LBW has been shown to increase the risk of later hypertension [41]. In meta-analyses, the mean systolic BP was 2.5 (95% CI: 1.7–3.3) mm Hg higher in subjects born preterm than at term [41] and 2.3 mm Hg higher in subjects with BW below 2500 g compared to those with a normal BW [42]. Since the heights of preterm born children of the present study were lower than their term-born peers, and BP values for children and adolescents rest on height percentiles, we expected they would have lower BP. In agreement with previous studies, we observed positive correlations between systolic BP and indices of body fat distribution [43]. However, despite an increased truncal fat mass, we found no difference in BP between preterm and term-born children. This may have been due to the limited size of the study.

### Growth and body composition in relation to prematurity, low birth weight and SGA

Apart from height, growth parameters were similar in preterm and term-born children. The preterm SGA children had the shortest stature, but the highest subscapular to triceps skinfold ratio, suggesting a relatively higher truncal fat mass. Similar observations have been made in many [44–46], but not all [47] previous studies investigation these issues. In a recent large study on 11 year old Belarussian children, those who were born SGA were reported to be thinner and shorter, and with less fat than children born AGA; however, that study did not include children with a BW <2500 gram [47].

In line with the DohAD concept, most epidemiological studies suggest that being born SGA is associated with higher percentages of body fat and truncal obesity in later childhood and adulthood [44–46]. The causal pathway might have altered fetal programming linking restricted fetal growth, risk of the metabolic syndrome and chronic diseases in adulthood [46]. Our finding of impaired renal function in preterm SGA children fit this hypothesis.

### Strengths and limitations of the study

This was a population-based study. It included three endogenous markers of renal function, including SDMA, which has not previously been evaluated in a preterm population. The attendance rate was high, and the recruitment of the control group followed a strict algorithm based on the ‘next-born-subject’ principle, minimizing risks of selection bias. The study design prescribed inclusion of all subjects born EP (GA < 28 weeks), but also those with ELBW (< 1000 gram) irrespective of GA. Thus, the results indicate that extreme prematurity, and in particular early intrauterine growth restriction, may be risk factors for early renal impairment and related health consequences. However, the results do not necessarily generalize to all preterm-born children. The low number of participants is an obvious weakness, impacting statistical power and opening for type I as well as type II statistical errors, although the size of the study population is comparable to that of most follow-up studies on children born EP/ELBW. Lack of data on urine albuminuria, urine albumin-creatinine ratio or 24 h ambulatory blood pressure, represent additional weaknesses of the study.

## Conclusion

Eleven year old EP/ELBW children, and in particular those born SGA, had poorer renal function than peers born at term. The findings indicate that being born preterm and SGA implies an increased risk of developing kidney disease later in life.

## Abbreviations

AGA: Appropriate weight for Gestational Age
BW: Birth Weight
EP: extremely preterm
ELBW: extremely low birth weight
G: Grams
GA: Gestational Age
IQR: Interquartile Range
LBW: low birth weight
SD: Standard Deviation
SGA: Small for Gestational Age
CRP: C-Reactive Protein
GFR: Glomerular Filtration Rate
BP: Blood Pressure
BMI: Body Mass Index

## References

[1] Wilcox AJ, Weinberg CR, Basso O. On the pitfalls of adjusting for gestational age at birth. American journal of epidemiology. 2011;174(9):1062–8. Epub 2011/09/29. 10.1093/aje/kwr230 ; PubMed Central PMCID: PMCPmc3243938. 21946386 PMC3243938

[2] WHO MoD, Partnership for Maternal, Newborn & Child Health, Save the Children. Born too soon: the global action report on preterm birth.

[3] Horbar JD, Carpenter JH, Badger GJ, Kenny MJ, Soll RF, Morrow KA, et al Mortality and neonatal morbidity among infants 501 to 1500 grams from 2000 to 2009. Pediatrics. 2012;129(6):1019–26. Epub 2012/05/23. 10.1542/peds.2011-3028 . 22614775

[4] Petrou S, Eddama O, Mangham L. A structured review of the recent literature on the economic consequences of preterm birth. Arch Dis Child Fetal Neonatal Ed. 2011;96(3):F225–32. Epub 2010/05/22. 10.1136/adc.2009.161117 . 20488863

[5] Luyckx VA, Bertram JF, Brenner BM, Fall C, Hoy WE, Ozanne SE, et al Effect of fetal and child health on kidney development and long-term risk of hypertension and kidney disease. Lancet. 2013;382(9888):273–83. 10.1016/S0140-6736(13)60311-6 . 23727166

[6] Carmody JB, Charlton JR. Short-term gestation, long-term risk: prematurity and chronic kidney disease. Pediatrics. 2013;131(6):1168–79. Epub 2013/05/15. 10.1542/peds.2013-0009 . 23669525

[7] Johansson S, Iliadou A, Bergvall N, Tuvemo T, Norman M, Cnattingius S. Risk of high blood pressure among young men increases with the degree of immaturity at birth. Circulation. 2005;112(22):3430–6. Epub 2005/11/23. 10.1161/CIRCULATIONAHA.105.540906 . 16301344

[8] Lackland DT, Bendall HE, Osmond C, Egan BM, Barker DJ. Low birth weights contribute to high rates of early-onset chronic renal failure in the Southeastern United States. Archives of internal medicine. 2000;160(10):1472–6. Epub 2000/05/29. . 10826460 10.1001/archinte.160.10.1472

[9] Li S, Chen SC, Shlipak M, Bakris G, McCullough PA, Sowers J, et al Low birth weight is associated with chronic kidney disease only in men. Kidney international. 2008;73(5):637–42. Epub 2007/12/21. 10.1038/sj.ki.5002747 . 18094674

[10] Plantinga LC, Tuot DS, Powe NR. Awareness of chronic kidney disease among patients and providers. Advances in chronic kidney disease. 2010;17(3):225–36. 10.1053/j.ackd.2010.03.002 ; PubMed Central PMCID: PMC2864779. 20439091 PMC2864779

[11] Hsu CY, Bansal N. Measured GFR as "gold standard"—all that glitters is not gold? Clinical journal of the American Society of Nephrology: CJASN. 2011;6(8):1813–4. Epub 2011/07/26. 10.2215/CJN.06040611 . 21784836

[12] Pottel H. Measuring and estimating glomerular filtration rate in children. Pediatric nephrology (Berlin, Germany). 2017;32(2):249–63. Epub 2016/04/27. 10.1007/s00467-016-3373-x . 27115887

[13] Inker LA, Schmid CH, Tighiouart H, Eckfeldt JH, Feldman HI, Greene T, et al Estimating glomerular filtration rate from serum creatinine and cystatin C. N Engl J Med. 2012;367(1):20–9. Epub 2012/07/06. 10.1056/NEJMoa1114248 ; PubMed Central PMCID: PMCPmc4398023. 22762315 PMC4398023

[14] Gao A, Cachat F, Faouzi M, Bardy D, Mosig D, Meyrat BJ, et al Comparison of the glomerular filtration rate in children by the new revised Schwartz formula and a new generalized formula. Kidney international. 2013;83(3):524–30. Epub 2012/12/21. 10.1038/ki.2012.388 . 23254901

[15] Rink N, Zappitelli M. Estimation of glomerular filtration rate with and without height: effect of age and renal function level. Pediatric nephrology (Berlin, Germany). 2015;30(8):1327–36. Epub 2015/04/10. 10.1007/s00467-015-3063-0 . 25854613

[16] Schwartz GJ, Munoz A, Schneider MF, Mak RH, Kaskel F, Warady BA, et al New equations to estimate GFR in children with CKD. Journal of the American Society of Nephrology: JASN. 2009;20(3):629–37. Epub 2009/01/23. 10.1681/ASN.2008030287 ; PubMed Central PMCID: PMCPmc2653687. 19158356 PMC2653687

[17] Uemura O, Honda M, Matsuyama T, Ishikura K, Hataya H, Yata N, et al Age, gender, and body length effects on reference serum creatinine levels determined by an enzymatic method in Japanese children: a multicenter study. Clinical and experimental nephrology. 2011;15(5):694–9. Epub 2011/04/21. 10.1007/s10157-011-0452-y . 21505953

[18] Delanaye P, Cavalier E, Pottel H. Serum Creatinine: Not So Simple! Nephron. 2017 10.1159/000469669 . 28441651

[19] Zaffanello M, Franchini M, Fanos V. Is serum Cystatin-C a suitable marker of renal function in children? Annals of clinical and laboratory science. 2007;37(3):233–40. Epub 2007/08/22. . 17709686

[20] Kielstein JT, Salpeter SR, Bode-Boeger SM, Cooke JP, Fliser D. Symmetric dimethylarginine (SDMA) as endogenous marker of renal function—a meta-analysis. Nephrology, dialysis, transplantation: official publication of the European Dialysis and Transplant Association—European Renal Association. 2006;21(9):2446–51. Epub 2006/06/13. 10.1093/ndt/gfl292 . 16766542

[21] Markestad T, Kaaresen PI, Ronnestad A, Reigstad H, Lossius K, Medbo S, et al Early death, morbidity, and need of treatment among extremely premature infants. Pediatrics. 2005;115(5):1289–98. Epub 2005/05/04. 10.1542/peds.2004-1482 . 15867037

[22] Leversen KT, Sommerfelt K, Ronnestad A, Kaaresen PI, Farstad T, Skranes J, et al Prediction of neurodevelopmental and sensory outcome at 5 years in Norwegian children born extremely preterm. Pediatrics. 2011;127(3):e630–8. Epub 2011/02/16. 10.1542/peds.2010-1001 . 21321031

[23] Skjaerven R, Gjessing HK, Bakketeig LS. Birthweight by gestational age in Norway. Acta obstetricia et gynecologica Scandinavica. 2000;79(6):440–9. Epub 2000/06/17. . 10857867

[24] Moreno LA, Fleta J, Mur L, Feja C, Sarria A, Bueno M. Indices of body fat distribution in Spanish children aged 4.0 to 14.9 years. Journal of pediatric gastroenterology and nutrition. 1997;25(2):175–81. . 9252904 10.1097/00005176-199708000-00008

[25] Ueland PM, Midttun O, Windelberg A, Svardal A, Skalevik R, Hustad S. Quantitative profiling of folate and one-carbon metabolism in large-scale epidemiological studies by mass spectrometry. Clin Chem Lab Med. 2007;45(12):1737–45. 10.1515/CCLM.2007.339 . 17963453

[26] Meyer K, Ueland PM. Targeted quantification of C-reactive protein and cystatin c and its variants by immuno-MALDI-MS. Analytical chemistry. 2014;86(12):5807–14. 10.1021/ac500704y . 24848523

[27] Luyckx VA, Bertram JF, Brenner BM, Fall C, Hoy WE, Ozanne SE, et al Effect of fetal and child health on kidney development and long-term risk of hypertension and kidney disease. The Lancet. 2013;382(9888):273–83. 10.1016/s0140-6736(13)60311-6 23727166

[28] Rodriguez MM, Gomez AH, Abitbol CL, Chandar JJ, Duara S, Zilleruelo GE. Histomorphometric analysis of postnatal glomerulogenesis in extremely preterm infants. Pediatric and developmental pathology: the official journal of the Society for Pediatric Pathology and the Paediatric Pathology Society. 2004;7(1):17–25. Epub 2004/07/17. 10.1007/s10024-003-3029-2 . 15255031

[29] Black MJ, Sutherland MR, Gubhaju L, Kent AL, Dahlstrom JE, Moore L. When birth comes early: effects on nephrogenesis. Nephrology (Carlton, Vic). 2013;18(3):180–2. Epub 2013/01/03. 10.1111/nep.12028 . 23279726

[30] Luyckx VA, Brenner BM. Birth weight, malnutrition and kidney-associated outcomes—a global concern. Nat Rev Nephrol. 2015;11(3):135–49. 10.1038/nrneph.2014.251 . 25599618

[31] Cheung YF, Wong KY, Lam BC, Tsoi NS. Relation of arterial stiffness with gestational age and birth weight. Arch Dis Child. 2004;89(3):217–21. Epub 2004/02/24. 10.1136/adc.2003.025999 ; PubMed Central PMCID: PMC1719813. 14977693 PMC1719813

[32] Willemsen RH, de Kort SW, van der Kaay DC, Hokken-Koelega AC. Independent effects of prematurity on metabolic and cardiovascular risk factors in short small-for-gestational-age children. J Clin Endocrinol Metab. 2008;93(2):452–8. Epub 2007/11/22. doi: jc.2007-1913 [pii] 10.1210/jc.2007-1913 . 18029462

[33] Hinchliffe SA, Lynch MR, Sargent PH, Howard CV, Van Velzen D. The effect of intrauterine growth retardation on the development of renal nephrons. British journal of obstetrics and gynaecology. 1992;99(4):296–301. Epub 1992/04/01. . 1581274 10.1111/j.1471-0528.1992.tb13726.x

[34] Ortiz LA, Quan A, Zarzar F, Weinberg A, Baum M. Prenatal dexamethasone programs hypertension and renal injury in the rat. Hypertension (Dallas, Tex: 1979). 2003;41(2):328–34. Epub 2003/02/08. ; PubMed Central PMCID: PMCPmc4127977. 12574103 10.1161/01.hyp.0000049763.51269.51 PMC4127977

[35] Fanos V, Antonucci R, Zaffanello M, Mussap M. Nenatal drug induced nephrotoxicity: old and next generation biomarkers for early detection and management of neonatal drug-induced nephrotoxicity, with special emphasis on uNGAL and on metabolomics. Current medicinal chemistry. 2012;19(27):4595–605. Epub 2012/08/11. . 22876902 10.2174/092986712803306439

[36] Hughson M, Farris AB 3rd, Douglas-Denton R, Hoy WE, Bertram JF. Glomerular number and size in autopsy kidneys: the relationship to birth weight. Kidney international. 2003;63(6):2113–22. Epub 2003/05/20. 10.1046/j.1523-1755.2003.00018.x 12753298

[37] Vollsaeter M, Skromme K, Satrell E, Clemm H, Roksund O, Oymar K, et al Children Born Preterm at the Turn of the Millennium Had Better Lung Function Than Children Born Similarly Preterm in the Early 1990s. PloS one. 2015;10(12):e0144243 Epub 2015/12/08. 10.1371/journal.pone.0144243 ; PubMed Central PMCID: PMCPmc4671691. 26641080 PMC4671691

[38] Ronkainen E, Dunder T, Kaukola T, Marttila R, Hallman M. Intrauterine growth restriction predicts lower lung function at school age in children born very preterm. Arch Dis Child Fetal Neonatal Ed. 2016;101(5):F412–7. Epub 2016/01/24. 10.1136/archdischild-2015-308922 . 26802110

[39] Brooks ER, Langman CB, Wang S, Price HE, Hodges AL, Darling L, et al Methylated arginine derivatives in children and adolescents with chronic kidney disease. Pediatric nephrology. 2009;24(1):129–34. 10.1007/s00467-008-0972-1 . 18830716

[40] Soeby K, Jensen PB, Werge T, Sorensen S. Mining of hospital laboratory information systems: a model study defining age- and gender-specific reference intervals and trajectories for plasma creatinine in a pediatric population. Clin Chem Lab Med. 2015 10.1515/cclm-2014-0949 . 25719320

[41] de Jong F, Monuteaux MC, van Elburg RM, Gillman MW, Belfort MB. Systematic review and meta-analysis of preterm birth and later systolic blood pressure. Hypertension. 2012;59(2):226–34. 10.1161/HYPERTENSIONAHA.111.181784 ; PubMed Central PMCID: PMC3266458. 22158643 PMC3266458

[42] Mu M, Wang SF, Sheng J, Zhao Y, Li HZ, Hu CL, et al Birth weight and subsequent blood pressure: a meta-analysis. Archives of cardiovascular diseases. 2012;105(2):99–113. 10.1016/j.acvd.2011.10.006 . 22424328

[43] Freedman DS, Dietz WH, Srinivasan SR, Berenson GS. Risk factors and adult body mass index among overweight children: the Bogalusa Heart Study. Pediatrics. 2009;123(3):750–7. 10.1542/peds.2008-1284 . 19254998

[44] Elia M, Betts P, Jackson DM, Mulligan J. Fetal programming of body dimensions and percentage body fat measured in prepubertal children with a 4-component model of body composition, dual-energy X-ray absorptiometry, deuterium dilution, densitometry, and skinfold thicknesses. Am J Clin Nutr. 2007;86(3):618–24. 10.1093/ajcn/86.3.618 . 17823425

[45] Dolan MS, Sorkin JD, Hoffman DJ. Birth weight is inversely associated with central adipose tissue in healthy children and adolescents. Obesity (Silver Spring). 2007;15(6):1600–8. 10.1038/oby.2007.189 . 17557998

[46] Biosca M, Rodriguez G, Ventura P, Samper MP, Labayen I, Collado MP, et al Central adiposity in children born small and large for gestational age. Nutr Hosp. 2011;26(5):971–6. 10.1590/S0212-16112011000500008 . 22072340

[47] Kramer MS, Martin RM, Bogdanovich N, Vilchuk K, Dahhou M, Oken E. Is restricted fetal growth associated with later adiposity? Observational analysis of a randomized trial. Am J Clin Nutr. 2014;100(1):176–81. 10.3945/ajcn.113.079590 ; PubMed Central PMCID: PMC4144097. 24787489 PMC4144097

